# Publisher Correction: Abrupt emissions reductions during COVID-19 contributed to record summer rainfall in China

**DOI:** 10.1038/s41467-022-29156-0

**Published:** 2022-03-11

**Authors:** Yang Yang, Lili Ren, Mingxuan Wu, Hailong Wang, Fengfei Song, L. Ruby Leung, Xin Hao, Jiandong Li, Lei Chen, Huimin Li, Liangying Zeng, Yang Zhou, Pinya Wang, Hong Liao, Jing Wang, Zhen-Qiang Zhou

**Affiliations:** 1 Jiangsu Key Laboratory of Atmospheric Environment Monitoring and Pollution Control, Jiangsu Collaborative Innovation Center of Atmospheric Environment and Equipment Technology, School of Environmental Science and Engineering, Nanjing University of Information Science and Technology, Nanjing, Jiangsu China; 2 Atmospheric Sciences and Global Change Division, Pacific Northwest National Laboratory, Richland, WA USA; 3 Frontier Science Centre for Deep Ocean Multispheres and Earth System and Physical Oceanography Laboratory, Ocean University of China, Qingdao, China; 4 Qingdao National Laboratory for Marine Science and Technology (QNLM), Qingdao, China; 5 Collaborative Innovation Center on Forecast and Evaluation of Meteorological Disasters/Key Laboratory of Meteorological Disaster, Ministry of Education, Nanjing University for Information Science and Technology, Nanjing, Jiangsu China; 6 Tianjin Key Laboratory for Oceanic Meteorology, Tianjin Institute of Meteorological Science, Tianjin, China; 7 Department of Atmospheric and Oceanic Sciences and Institute of Atmospheric Sciences, Fudan University, Shanghai, China

**Keywords:** Atmospheric chemistry, Atmospheric chemistry

Correction to: *Nature Communications* (2022) **13**:1-7; 10.1038/s41467-022-28537-9, published online 18 February 2022.

In this article the affiliation details for Author L. Ruby Leung were incorrectly given as ‘Qingdao National Laboratory for Marine Science and Technology (QNLM), Qingdao, China’ but should have been ‘Atmospheric Sciences and Global Change Division, Pacific Northwest National Laboratory, Richland, WA, USA’. The original article has been corrected.

